# Supplementary material for: The benefits and harms of open notes in mental health: A Delphi survey of international experts
Source: PLoS One. 2021 Oct 13;16(10):e0258056. doi: 10.1371/journal.pone.0258056 (PMC8513879; doi:10.1371/journal.pone.0258056)
Supplement: S2 Appendix — (PDF) [file pone.0258056.s002.pdf]

Beth Israel Deaconess Medical Center

HARVARD MEDICAL SCHOOL TEACHING HOSPITAL

WELCOME

DELPHI CONSENSUS POLL
SHARING MENTAL HEALTH NOTES

- ROUND 2 -

OpenNotes®

INFORMATION

THANK YOU for your taking part in Round One of this Delphi Poll on the impact of open notes in mental health. We were delighted with the response rate, and appreciate you taking the time to provide your insights. Your participation in Round Two will help us to further refine the collective consensus from views gathered in Round One.

Round Two of the survey involves short close-ended questions. Because of the volume of responses, we are focusing only on questions about the benefits and harms of open notes in mental health. These questions were generated from the combined responses in Round One. **The survey has been pre-tested and should take around 10 minutes and no more than 15 minutes to complete.**

In the third and final round, which will be shorter, only answers with less agreement among participants will again be sent out anonymously. We anticipate Round Three will take 5 minutes to complete.

Thank you for lending us your expertise. It will not be possible to complete this study without you.

OUR TEAM

We appreciate your interest in participating in this online research survey. We are a team of researchers based at OpenNotes, Beth Israel Deaconess Medical Center, Harvard Medical School, Boston, USA; Digital Psychiatry, Department of Psychiatry, Deaconess Medical Center, Harvard Medical School, Boston, USA; the Department of and Children's Health, Uppsala University, Sweden; and the School of Psychology, University of Plymouth, UK.

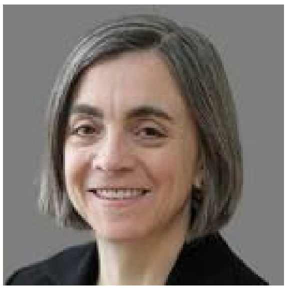

Dr. Catherine DesRoches

OpenNotes
Harvard Medical School

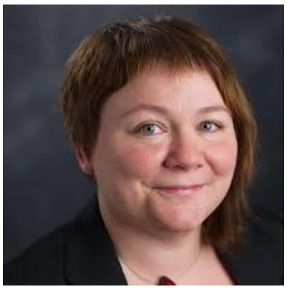

Dr. Maria Hägglund

OpenNotes
Uppsala University

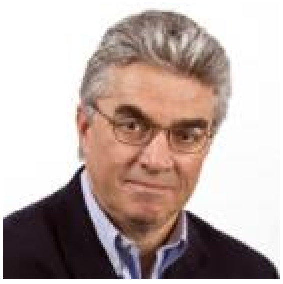

Dr. John Santa

Harvard
Medical School

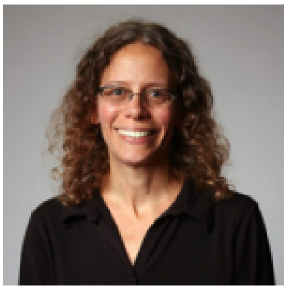

Deborah Wachenheim

OpenNotes
Harvard Medical School

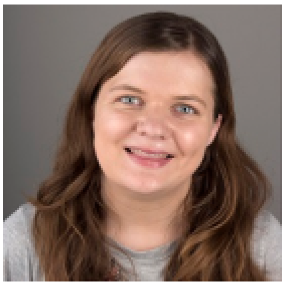

Kendall Harcourt

OpenNotes
Harvard Medical School

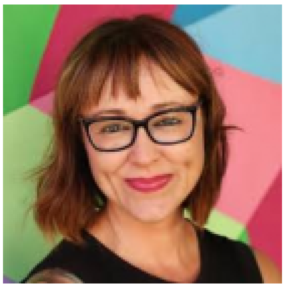

Liz Salmi

OpenNotes
Harvard Medical School

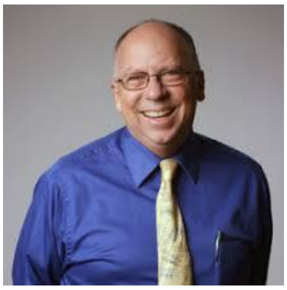

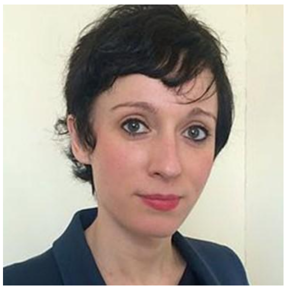

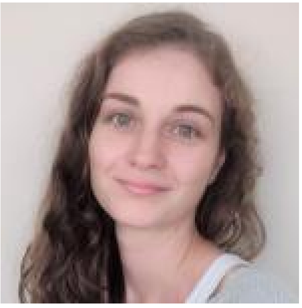

Dr. Stephen O'Neill
OpenNotes
Harvard Medical School

Dr. Charlotte Blease
OpenNotes
Harvard Medical School

Anna Kharko
University of Plymouth

PREAMBLE

ABOUT THE STUDY

TIME & COMMITMENT OF PARTICIPANTS

Round 2 will close at midnight EST on October 16th, 2020.

We will ensure that there is adequate time for panelists to provide their responses between rounds. Although this survey is not very demanding of time, the quality of Delphi Polls is dependent on a high level of participation between rounds. Therefore,

should you be willing to participate, we request that you consider whether you can commit to responding to each of the three survey rounds.

All the data is completely anonymous. This study has been given ethical approval by Beth Israel Deaconess Medical Center, Boston, and the University of Plymouth, UK. The reference number for this study is Protocol #: 2020P000218.

DO I HAVE TO TAKE PART?

Participation is voluntary. You may withdraw at any point during the questionnaire for any reason, before submitting your answers, by closing the browser.

HOW WILL MY DATA BE USED?

The data we gather will be stored in a password-protected file and will be used to inform future academic publications. All questions are optional. The data will be stored for a minimum of ten years after publication or public release. Delphi panelists will also be asked whether they wish to remain anonymous or agree to their name being published in a journal article upon which the aggregate data will be based. It will not be possible to link data to individual participants.

WHO WILL HAVE ACCESS TO MY DATA?

Qualtrics (https://www.qualtrics.com/about/) is the data controller. You can read about their security policies here: https://www.qualtrics.com/security-statement/. The information will not be shared with anyone, and your name will be replaced by a numerical ID in all data analysis. Only selected members of the study team will be given access to the anonymous data for monitoring and/or audit of the study to ensure we are complying with guidelines, or as otherwise required by law.

WHAT IF THERE IS A PROBLEM?

If you have a concern about any aspect of this project, please speak to the PI Dr Cait DesRoches (cdesroch@bidmc.harvard.edu) telephone 001.617.975.7612, or Dr Charlotte Blease (cblease@bidmc.harvard.edu) who will do their best to answer your

query. The researcher should acknowledge your concern within 10 working days and give you an indication of how they intend to deal with it. If you have any complaints about this survey, please contact HSPO on 001.617.975.8500

By signing this form, you agree that you have read and understood your rights and provide consent to participate in this research.

- I have read and understood the study information and give my consent to continue.
- I do not wish to continue.

ABOUT YOU

ABOUT YOU

Did you complete Round 1 of this survey?

- Yes
- No

First Name & Last Name

Email

BENEFITS

BENEFITS TO PATIENTS

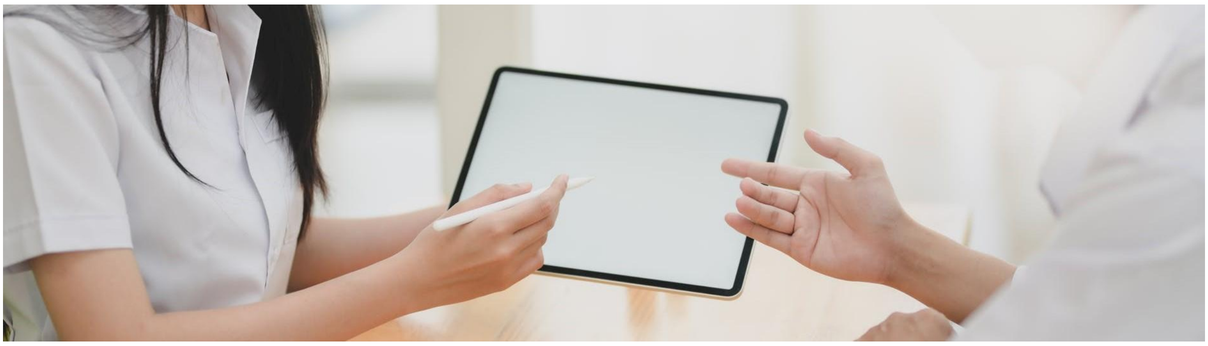

The following questions ask for **your expert opinions** about the benefits of patients receiving online access to their mental health notes. Unless otherwise stated, we interpret 'mental health notes' to refer to any clinical notes written about a patient's mental health including notes written in psychiatry, clinical psychology, social work, psychotherapy contexts.

Access to mental health notes will help patients to ...

	strongly disagree	moderately disagree	somewhat disagree	neutral	somewhat agree	moderately agree	strongly agree

	strongly disagree	moderately disagree	somewhat disagree	neutral	somewhat agree	moderately agree	strongly agree
correct errors in notes.	○	○	○	○	○	○	○
correct clinician misinterpretations	○	○	○	○	○	○	○
correct errors in how their social history is documented.	○	○	○	○	○	○	○
share their notes with other clinicians improving continuity of care .	○	○	○	○	○	○	○

Patient access to mental health notes will ...

	strongly disagree	moderately disagree	somewhat disagree	neutral	somewhat agree	moderately agree	strongly agree
help to close the feedback loop on care.	○	○	○	○	○	○	○
help to demystify psychotherapy.	○	○	○	○	○	○	○
prompt patients to research more about their healthcare.	○	○	○	○	○	○	○

Patient access to mental health notes will improve ...

	strongly disagree	moderately disagree	somewhat disagree	neutral	somewhat agree	moderately agree	strongly agree

	strongly disagree	moderately disagree	somewhat disagree	neutral	somewhat agree	moderately agree	strongly agree
clinical outcomes.	○	○	○	○	○	○	○
quality of care.	○	○	○	○	○	○	○
treatment processes.	○	○	○	○	○	○	○
mutual understanding between patients and clinicians.	○	○	○	○	○	○	○
patient-clinician goal-alignment.	○	○	○	○	○	○	○
patient-clinician communication.	○	○	○	○	○	○	○
the therapeutic alliance.	○	○	○	○	○	○	○

Access to mental health notes will improve patient understanding about their ...

	strongly disagree	moderately disagree	somewhat disagree	neutral	somewhat agree	moderately agree	strongly agree
care plan.	○	○	○	○	○	○	○
diagnosis / mental health condition.	○	○	○	○	○	○	○
health changes over time.	○	○	○	○	○	○	○
the rationale for their treatments.	○	○	○	○	○	○	○

Access to mental health notes will improve patient recall ...

	strongly disagree	moderately disagree	somewhat disagree	neutral	somewhat agree	moderately agree	strongly agree
about what was communicated during the visit.	○	○	○	○	○	○	○
especially among patients with memory or cognitive problems.	○	○	○	○	○	○	○
of homework between visits.	○	○	○	○	○	○	○

Access to mental health notes will improve patient ...

	strongly disagree	moderately disagree	somewhat disagree	neutral	somewhat agree	moderately agree	strongly agree
satisfaction with care.	○	○	○	○	○	○	○
trust in clinicians.	○	○	○	○	○	○	○
sense of control of their health.	○	○	○	○	○	○	○
sense of privacy over their health information.	○	○	○	○	○	○	○
responsibility for their healthcare.	○	○	○	○	○	○	○

	strongly disagree	moderately disagree	somewhat disagree	neutral	somewhat agree	moderately agree	strongly agree
awareness over current medications.	○	○	○	○	○	○	○
preparation for visits.	○	○	○	○	○	○	○

Access to mental health notes will improve patient adherence to ...

	strongly disagree	moderately disagree	somewhat disagree	neutral	somewhat agree	moderately agree	strongly agree
treatment plans.	○	○	○	○	○	○	○
medications.	○	○	○	○	○	○	○

Access to mental health notes will increase ...

	strongly disagree	moderately disagree	somewhat disagree	neutral	somewhat agree	moderately agree	strongly agree
the depth of patient-clinician dialogue during visits.	○	○	○	○	○	○	○
shared decision-making.	○	○	○	○	○	○	○
adoption of patient-centered language in clinicians' documentation.	○	○	○	○	○	○	○

	strongly disagree	moderately disagree	somewhat disagree	neutral	somewhat agree	moderately agree	strongly agree
the likelihood patients will ask relevant questions at visits.	◯	◯	◯	◯	◯	◯	◯
efficiency in patient care.	◯	◯	◯	◯	◯	◯	◯
economic efficiencies in healthcare.	◯	◯	◯	◯	◯	◯	◯

Access to mental health notes will diminish ...

	strongly disagree	moderately disagree	somewhat disagree	neutral	somewhat agree	moderately agree	strongly agree
problematic power imbalances between clinicians and patients.	◯	◯	◯	◯	◯	◯	◯
the use of disrespectful or derogatory language in notes.	◯	◯	◯	◯	◯	◯	◯
patient anxiety about what clinicians write about them.	◯	◯	◯	◯	◯	◯	◯
patient feelings of stigmatization.	◯	◯	◯	◯	◯	◯	◯

Patient access to mental health notes will improve family / friend / caregiver ...

	strongly disagree	moderately disagree	somewhat disagree	neutral	somewhat agree	moderately agree	strongly agree
trust in clinicians.	○	○	○	○	○	○	○
recall about patient appointments.	○	○	○	○	○	○	○

Do you have any other comments about benefits to patients?

HARMS

HARMS TO PATIENTS

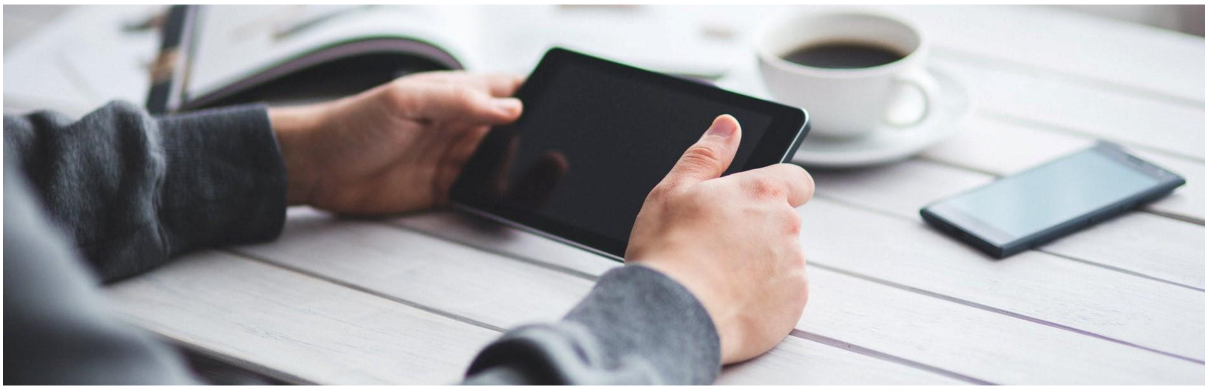

The following questions ask for **your expert opinions** about the harms of patients receiving online access to their mental health notes. Unless otherwise stated, we interpret 'mental health notes' to refer to any clinical notes written about a patient's mental health including notes written in psychiatry, clinical psychology, social work, psychotherapy contexts.

As a result of opening mental health notes there will be ...

	strongly disagree	moderately disagree	somewhat disagree	neutral	somewhat agree	moderately agree	strongly agree
no major harms to patients.	○	○	○	○	○	○	○
few harms to patients.	○	○	○	○	○	○	○
harms if notes are written without intention to share with patients.	○	○	○	○	○	○	○
harms if patients lack guidance on reading notes.	○	○	○	○	○	○	○
an increase in patients demanding changes to their notes.	○	○	○	○	○	○	○

Harm is more likely if patients ...

	strongly disagree	moderately disagree	somewhat disagree	neutral	somewhat agree	moderately agree	strongly agree
can access their notes immediately after a visit.	O	O	O	O	O	O	O

Hiding mental health / psychotherapy notes ...

	strongly disagree	moderately disagree	somewhat disagree	neutral	somewhat agree	moderately agree	strongly agree
from patients will result in greater harm.	O	O	O	O	O	O	O
will lead to greater patient stigmatization.	O	O	O	O	O	O	O

Patients who disagree with their diagnosis ...

	strongly disagree	moderately disagree	somewhat disagree	neutral	somewhat agree	moderately agree	strongly agree
will be less likely to attend visits.	O	O	O	O	O	O	O

Clinicians will ...

	strongly disagree	moderately disagree	somewhat disagree	neutral	somewhat agree	moderately agree	strongly agree

	strongly disagree	moderately disagree	somewhat disagree	neutral	somewhat agree	moderately agree	strongly agree
spend less time in patient visits.	○	○	○	○	○	○	○

Clinicians will be less detailed / accurate in documenting ...

	strongly disagree	moderately disagree	somewhat disagree	neutral	somewhat agree	moderately agree	strongly agree
negative aspects of patient relationship.	○	○	○	○	○	○	○
patients' personalities.	○	○	○	○	○	○	○
symptoms of paranoia in patients.	○	○	○	○	○	○	○
substance abuse disorders.	○	○	○	○	○	○	○
differential diagnoses.	○	○	○	○	○	○	○

Access to mental health notes will be detrimental to ...

	strongly disagree	moderately disagree	somewhat disagree	neutral	somewhat agree	moderately agree	strongly agree
the therapeutic alliance.	○	○	○	○	○	○	○

	strongly disagree	moderately disagree	somewhat disagree	neutral	somewhat agree	moderately agree	strongly agree
patient adherence to treatment plans.	○	○	○	○	○	○	○
clinician honesty in therapy sessions.	○	○	○	○	○	○	○

Access to mental health notes will increase ...

	strongly disagree	moderately disagree	somewhat disagree	neutral	somewhat agree	moderately agree	strongly agree
patient symptoms of mental illnesses.	○	○	○	○	○	○	○
patient threats towards clinicians.	○	○	○	○	○	○	○
patient violence towards clinicians.	○	○	○	○	○	○	○

When accessing their mental health notes patients will be confused by ...

	strongly disagree	moderately disagree	somewhat disagree	neutral	somewhat agree	moderately agree	strongly agree
psychiatric terms.	○	○	○	○	○	○	○

	strongly disagree	moderately disagree	somewhat disagree	neutral	somewhat agree	moderately agree	strongly agree
clinical abbreviations.	○	○	○	○	○	○	○

Mental health patients will be offended by ...

	strongly disagree	moderately disagree	somewhat disagree	neutral	somewhat agree	moderately agree	strongly agree
psychiatric terms.	○	○	○	○	○	○	○

Patients with the following conditions / symptoms will be unable to understand their mental health notes ...

	strongly disagree	moderately disagree	somewhat disagree	neutral	somewhat agree	moderately agree	strongly agree
psychosis.	○	○	○	○	○	○	○
delusional disorders.	○	○	○	○	○	○	○
paranoia.	○	○	○	○	○	○	○
violent patients.	○	○	○	○	○	○	○
personality disorders.	○	○	○	○	○	○	○
patients who are hospitalized for mental illness.	○	○	○	○	○	○	○

Patients with the following conditions / symptoms will be harmed from accessing their notes ...

	strongly disagree	moderately disagree	somewhat disagree	neutral	somewhat agree	moderately agree	strongly agree
bipolar disorder disorders.	○	○	○	○	○	○	○
major depressive disorders.	○	○	○	○	○	○	○
personality disorders.	○	○	○	○	○	○	○
patients who are suicidal.	○	○	○	○	○	○	○
patients with obsessive conditions.	○	○	○	○	○	○	○
eating disorders.	○	○	○	○	○	○	○

Too much detail in notes ...

	strongly disagree	moderately disagree	somewhat disagree	neutral	somewhat agree	moderately agree	strongly agree
will re-traumatize patients.	○	○	○	○	○	○	○

As a result of reading their mental health notes, a minority of patients will ...

	strongly disagree	moderately disagree	somewhat disagree	neutral	somewhat agree	moderately agree	strongly agree
be distressed.	○	○	○	○	○	○	○
feel ashamed.	○	○	○	○	○	○	○
feel insulted.	○	○	○	○	○	○	○
feel alienated by clinical language.	○	○	○	○	○	○	○
experience cyberchondria.	○	○	○	○	○	○	○

Perceived misunderstandings in mental health notes will cause patients to be ...

	strongly disagree	moderately disagree	somewhat disagree	neutral	somewhat agree	moderately agree	strongly agree
frustrated.	○	○	○	○	○	○	○
angry.	○	○	○	○	○	○	○

Patients in domestic abuse situations ...

	strongly disagree	moderately disagree	somewhat disagree	neutral	somewhat agree	moderately agree	strongly agree
will be less candid in visits.	○	○	○	○	○	○	○
will be at increased harm.	○	○	○	○	○	○	○

Do you have any other comments about harms to patients?

COMMENTS

COMMENTS

Do you have any other comments about sharing online mental health notes with patients?

Powered by Qualtrics
